# Parenteral Nutrition-Associated Liver Disease: The Role of the Gut Microbiota

**DOI:** 10.3390/nu9090987

**Published:** 2017-09-07

**Authors:** Monika Cahova, Miriam Bratova, Petr Wohl

**Affiliations:** 1 Centre for Experimental Medicine, Institute for Clinical and Experimental Medicine, Department of Metabolism and Diabetes, Videnska 1958/9, 14021 Prague 4, Czech Republic; miriam.bratova@ikem.cz; 2 Centre of Diabetology, Institute for Clinical and Experimental Medicine, Department of Metabolism and Diabetes, Videnska 1958/9, 14021 Prague 4, Czech Republic; petr.wohl@ikem.cz

**Keywords:** Parenteral nutrition, microbiota, PNALD, intestinal permeability, gut-associated immune system, bile acids, FXR signalling, pre/probiotics

## Abstract

Parenteral nutrition (PN) provides life-saving nutritional support in situations where caloric supply via the enteral route cannot cover the necessary needs of the organism. However, it does have serious adverse effects, including parenteral nutrition-associated liver disease (PNALD). The development of liver injury associated with PN is multifactorial, including non-specific intestine inflammation, compromised intestinal permeability, and barrier function associated with increased bacterial translocation, primary and secondary cholangitis, cholelithiasis, short bowel syndrome, disturbance of hepatobiliary circulation, lack of enteral nutrition, shortage of some nutrients (proteins, essential fatty acids, choline, glycine, taurine, carnitine, etc.), and toxicity of components within the nutrition mixture itself (glucose, phytosterols, manganese, aluminium, etc.). Recently, an increasing number of studies have provided evidence that some of these factors are directly or indirectly associated with microbial dysbiosis in the intestine. In this review, we focus on PN-induced changes in the taxonomic and functional composition of the microbiome. We also discuss immune cell and microbial crosstalk during parenteral nutrition, and the implications for the onset and progression of PNALD. Finally, we provide an overview of recent advances in the therapeutic utilisation of pro- and prebiotics for the mitigation of PN-associated liver complications.

## 1. Introduction

Parenteral nutrition (PN) provides life-saving nutritional support in situations where caloric supply via the enteral route is either not possible or cannot cover the necessary needs of the organism, e.g., preterm neonates with immature gut, perioperatively in patients requiring massive intestinal surgery, or in patients with short bowel syndrome (SBS). PN preserves lean body mass, supports immune functions, and reduces metabolic complications in patients who are otherwise unable to feed [1]. Nevertheless, PN does have serious adverse effects, one of which is the deterioration of liver function. PN-induced cholestasis generally refers to the onset of liver disease in the context of the administration of intravenous nutrition in patients with temporary or permanent intestinal failure. Other terms commonly used to describe the condition are parenteral nutrition-associated cholestasis (PNAC) and intestinal failure-associated liver disease (IFALD), but the most frequently used is parenteral nutrition-associated liver disease (PNALD) [2]. PNALD clinical manifestations—which range from steatosis, cholestasis, gallbladder sludge/stones, fibrosis, and cirrhosis—can occur separately or in combination [3,4]. Steatosis (defined as fat accumulation in hepatocytes) can present as mild to moderate elevations in liver function tests and is usually benign. Cholestasis results from impaired secretion or obstruction of bile, and is associated with elevations in alkaline phosphatase, gamma-glutamyl transferase, and conjugated bilirubin [5].

There are some differences between child and adult patients totally dependent on parenteral nutrition (TPN) with regard to the pathogenesis of PNALD. Cholestasis occurs in 40–60% of infants, steatosis in 40–55% of adults, while biliary sludge and cholelithiasis occur in both adults and children. Child TPN patients (premature infants) typically exhibit prematurity (impaired transsulfuration, lack of cystathionase, etc.) and bowel lengths <25 cm, while necrotising enterocolitis is frequently the cause of intestinal failure. In addition, the high-energy requirements (80–100 kcal/kg per day) for growth are associated with the rapid onset of PNALD within months. All of these factors make children patients significantly more susceptible to PNALD/IFALD. In adults, a history of PNALD is characterised by elevated liver enzymes and associated steatosis, which can last for years. Ensuing complications include steatohepatitis, cholestatic hepatitis, as well as fibrosis and cirrhosis. In contrast, histology findings in child-type PNALD are defined by cholestasis, portal fibrosis, peri-cellular fibrosis, bile duct proliferation, pigmented Kupffer cells and non-progressive cirrhosis. Risk factors in children include lack of taurine, excess of calories, excess of lipids (>3.5 g/kg/day, mainly omega-6 fatty acids), phytosterols, bowel inflammation, catheter infections, and an absence of the ileo-caecal valve. In adults, risk factors include lack of choline, excess of calories, excess of lipids >1 g/kg/day, phytosterols, bowel inflammation, small bowel bacterial overgrowth, and absence of the ileo-caecal valve. In both groups, sepsis is a significant complication. The detailed differences of the pathomechanisms that underlie liver damage in both PN-dependent paediatric and adult patients are beyond the scope of this paper. More information is available in some excellent recent reviews [6,7]. 

Although the pathogenesis of PNALD is undoubtedly a multifactorial phenomenon, the privileged role may be attributed to the impaired function of the intestine. It harbours most of the immune cells in the body and represents the largest area for contact with antigens from the environment. The gut microbiome plays an essential role in intestinal development and homeostasis maintenance [8]. In humans, the gut microbiome is composed of approximate 1000 species and it is estimated that their genomes exceed the human genome by more than one hundred-fold. This represents a major stimulus to the immune system and facilitates the performance of many physiological functions [9]. The gut microbiota is the most abundant cohort of antigen-presenting cells. Based on the above facts, it is conceivable that the radical alteration of gut microbiome composition and function as a result of switching to total parenteral nutrition could lead to detrimental effects on the intestine and significantly contribute to PNALD development.

## 2. PN and the Gut Microbiota

Data on the gut microbiota in the context of PN have been obtained under three different group settings: animal models (mouse, rat, piglet), neonate babies (most of them premature), and adult patients who have lost a substantial portion of functional gut tissue and cannot feed themselves via the enteral route. Although PN is present in all cases, each of these groups has specific features that strongly influence microbiome composition (Table 1).

### 2.1. Adult Animal Models

In rats, Hodin et al. demonstrated that after 14 days of PN, total bacterial numbers per gram of luminal content did not differ between PN-fed and control rats; however, the composition of the bacterial population significantly changed. *Firmicutes* abundance dropped while the abundance of *Bacteroidetes* did not differ between groups. Consequently, the proportional representation of these two phyla in PN rats significantly shifted in favour of *Bacteroidetes* [10]. In a mouse model (5 days of PN), Miyasaka demonstrated that at the phylum level, the vast majority of mucosa-associated bacteria in the small bowels of control mice were *Firmicutes*. However, in the PN group, the dominant phyla were *Proteobacteria* and *Bacteroidetes*. At the genus level, PN mice had more bacteria in the genera *Salmonella, Escherichia, Proteus,* and *Bacteroides*. These genera are often associated with clinical infections, potentially indicating the development of a pathological state within the intestinal microbial community. Furthermore, enteral nutrient deprivation resulted in a loss of diversity in the large and small intestines [11]. Similar results indicating a shift from *Firmicutes* to *Bacteroides* have also been reported by an independent group [12]. A common feature of all these models is the overall deprivation of enteral nutrition for the entire duration of the experiments.

### 2.2. Neonates: Humans

In preterm neonates, the immature gut is much more prone to insults resulting from reduced intestinal motility, inappropriate immune responses, decreased protective gastrointestinal secretions, reduced digestive and absorptive function, and increased intestinal epithelial permeability [22]. In a prospective two-centre study, Parm et al. compared the effect of enteral-versus-parenteral feeding on the pattern of gut colonisation in preterm neonates at risk of late-onset sepsis and necrotising enterocolitis. PN was associated with the reduced acquisition of both Gram-positive and Gram-negative colonising microorganisms. *Candida albicans* colonisation was more frequent in neonates receiving PN. Despite greater mucosal colonisation by potentially pathogenic microorganisms (e.g., *Enterobacteriaceae* and *Enterococcus*), an enteral feeding regimen was associated with lower odds of late-onset sepsis and mortality in premature neonates than a PN regimen [17]. *Enterococcus faecalis* is the main immune modulator among human intestinal lactic acid bacteria, and is able to downregulate the expression of the host immune genes that participate in inflammation [23]. Thus, in the context of the naïve gut, colonisation with some *Enterococci* may be beneficial due to the suppression of specific toll-like receptors (TLR)-signalling pathways. Nevertheless, the conclusions drawn by Parm et al. are limited in that their study only included individuals under third-level neonatal intensive care, with all participants receiving at least one but usually more antibiotics, all of which may have significantly influenced gut microflora composition. 

### 2.3. Neonates: Animal Models

In controlled animal model experiments that investigate the effect of PN on gut colonisation in neonates, enterally fed piglets exhibited a higher bacterial diversity, higher concentrations of bacteria (CFU/g), and increased colonisation of all segments of the intestinal tract compared to PN pigs. Translocation of bacteria from the intestinal tract to tissues or blood was similar in both groups. PN-treated piglets were at higher risk of colonisation by toxin-expressing strains of *C. difficile* [14]. Using the same model but a different method of bacterial taxa identification (16S rRNA NGS sequencing versus DGGE analysis), Deplancke found that the bacterial community structure was equally complex in the ilea of enterally and parenterally fed piglets; however, profiles clustered according to the mode of nutrition. The opportunistic pathogen *C. perfringens,* as well as mucus-associated bacteria, were specifically enriched in the guts of animals dependent on PN [15]. Bacteria capable of using sulphated monosaccharides were also more abundant in PN samples. 

A recently published study by Lavallee et al. demonstrated that not only PN *per se* but also the type of lipid constituent affects microbiome composition in newborns. As expected, the gut microbial composition of PN-dependent piglets differed from those fed with sow milk, but the microbiota further clustered according to ω-3 or ω-6 PUFA content in the nutrition mixture. Piglets fed with ω-3 PUFA-rich PN were more similar to the sow milk-fed group than those administered ω-6 PUFA. The group with prevailing ω-6 PUFA on PN showed a specific and significant increase in *Parabacteroides*, while the ω-3 group showed an increase in *Enterobacteriaceae* [16]. The limitation of this study is that the antibiotic treatment was administered only to piglets exhibiting signs of sepsis, but not globally to the whole cohort. Nevertheless, this innovative study points out an unexpected aspect of PN, namely the interplay of the gut microbiota and lipid components of the nutrition mixture. 

### 2.4. Patients with Small Intestinal Resections: Fed vs. Enterally Deprived Portions of the Intestine

The common denominator for all of the above therapeutic or experimental settings is the absence of enteral nutrition, which leaves the intestinal microbiota in a state of acute nutrient withdrawal. No animal study comparing the effect of PN alone or PN in combination with enteral nutrition has been carried out. Ralls et al. published an interesting study comparing microbial diversity and differences in microbial characteristics in enterally fed and enterally deprived portions of the small bowel obtained after surgical resection [18]. The restraint of this study was the low number of samples used and the highly heterogeneous population of the patients. However, some valuable implications can be derived. Although only a partial stratification of microbial communities between fed and enterally deprived groups was found, some groups tended to expand in the fed group (*Staphylococcus, Pseudomonas, Campylobacter, Propionibacterium, Chryseomonas*) in comparison to others in the enterally deprived group (*Enterobacter, Shigella, Klebsiella and Fusobacterium*). A close correlation was identified in patients with low levels of enteric microbial diversity and those who developed post-operative enteric-derived infections.

### 2.5. Adult SBS Patients: The Specific Intestinal Environment

Patients diagnosed with short bowel syndrome (SBS) represent a specific group that are dependent on total parenteral nutrition. SBS occurs in patients with an extensive resection of the short bowel, leaving less than 150 cm. These patients suffer from severely decreased absorption capacity for water, electrolytes, and nutrients, while intravenous supplementation is required to maintain vital functions [24]. General adaptations following intestinal resection include compensatory hyperphagia, mucosal remodelling of the remaining part of the intestine and major modifications of the microbiota [25]. In contrast to previously described patient groups and animal models, these subjects do intake food per os, whereby the residual lumen of the gut is supplied with abundant but poorly digested substrates. In addition to the excessive nutrient delivery, the gut ecological system of SBS patients is altered in several other ways. Faecal pH has been shown to be lower in patients than in controls, with mean faecal pH at approx. 5.6 in type II SBS patients in comparison to the normal laboratory range of pH (pH = 6 to pH = 7) [19]. Acidic pH creates a specific environment that favours low pH-tolerant microorganisms [26]. In SBS patients, it is possible that because of the short length of the remnant small intestine and colon, the level of oxygen becomes too high, and thus anaerobic bacteria are discriminated in favour of more tolerant facultative anaerobes. The disruption of enterohepatic circulation may result in disturbed bile acid metabolism and a preference for microbiota that are tolerant to bile acids. 

### 2.6. Adult SBS Patients: Microbiota Composition

Original studies that describe the microbiome in SBS patients are remarkably consistent in their findings. The common feature of the SBS microbiota is significantly reduced α-diversity when compared with healthy controls [21,27,28], which positively correlates with remaining small bowel length [21] and PN dependence duration [28]. SBS patients harbour a specific faecal microbiota that is enriched in the *Lactobacillus/Leuconostoc* group and depleted in anaerobic microorganisms, especially those of the *Clostridiaceae* family [19,20]. A characteristic feature of this microbiota is its high abundance of *Proteobacteria*, especially *Enterobacteriaceae* [21,28]. The expansion of *Proteobacteria* may be linked to altered nutrition supply, as *Proteobacteria* can metabolise broader classes of substrates (including amino acids) and are therefore more flexible than other phyla. The decrease of *Bacteroidetes* has been reported both for mucosa [19] and luminal content [28]. The important feature of the SBS microbiome is the alteration of the *Firmicutes* community. *Firmicutes* is a dominant phylum of the healthy gut microbiota. In SBS patients, this phylum is still highly abundant but some important families of butyrate-producers, such as *Lachnospiraceae, Ruminococcaceae* and others, are almost entirely absent [21]. 

The severe dysbiosis found in SBS patients has a significant impact on the faecal metabolome and seriously affects the host metabolism. The bioconversion of macromolecules into metabolites is carried out by the bacteria of various functional groups, resulting in metabolic trophic chains. In SBS, the trophic chains and fermentation end-products are produced by the lactobiota, and are thus different from those produced by the healthy microbiota. The dominance of the carbohydrate-fermenting *Lactobacillus/Leuconostoc* taxa and the depletion of lactate-consuming anaerobic bacteria result in the excessive formation of both d- and l-lactate. Mayeur et al. [27] demonstrated that the individual lactic acid bacteria composition predicts whether or not a particular SBS patient will accumulate d-lactate in stools. In healthy humans, the lactate produced by the gut microbiota is not accumulated in faeces but it is readily absorbed by intestinal cells or converted to other metabolites, particularly the short-chain fatty acids (SCFA) [29,30]. Furthermore, in healthy humans, d-lactate is efficiently metabolised in the liver, and is thus eliminated from circulation. SBS patients who often suffer from compromised liver function are at an increased risk of lactate acidosis and d-lactate encephalopathy. Another consequence of the profound shift in microbiota composition, particularly the elimination of butyrate-producing anaerobes, is the significant decrease in SCFA production. This SCFA shortage may have multiple impacts on the gut immune system, intestinal wall integrity, and endocrine signalling.

## 3. PN and the Immune System

The gut microbiota profoundly influences the host immune system [31]. Numerous microbial products—including proteins, polysaccharides, and molecules that activate pattern recognition receptors—activate Toll-like receptors (TLRs) and NOD-like receptors (NODs) and stimulate the mucosal immune system [32]. The gut microbiota has an irreplaceable role in mucosal and systemic immunity maturation [33,34,35]. It also promotes a tolerogenic state in the intestinal mucosa (stimulation of Treg lymphocytes, attenuation of NF-κB signalling, etc.) [36,37,38,39,40], and instigates mechanisms that prevent bacterial overgrowth (induction of IgA secretion) [41,42,43,44]. Some commensals even produce targeted antimicrobial peptides themselves [45]. The healthy gut microbiota provides its host with a physical barrier to incoming pathogens and stimulates it to produce various antimicrobial compounds [31]. 

### 3.1. The Microbiota and TLR Signalling

The loss of diversity associated with prolonged PN administration has several adverse impacts on immune functions. The extinction of normally robust commensals facilitates the expansion of pathogenic strains due to the alleviation of competitive exclusion. For instance, the *Proteobacteria* found in the intestines of PN patients includes the opportunistic pathogens *E. coli*, *Salmonella*, *Yersinia*, *Helicobacter*, and *Vibrio*, all of which are commonly associated with infection [46]. Concurrently, some beneficial commensals that normally stimulate immunotolerance are underrepresented in the PN-associated microbiota. The shift in the intestinal microbiota (particularly the enrichment in *Proteobacteria* expressing many TLR ligands) and the activation of MyD88-dependent TLR signalling are suspected as causes of pro-inflammatory status in the PN-associated intestinal mucosa. Nevertheless, results obtained from mice models deficient in TLR signalling in intestinal epithelial cells are contradictory. Several authors have shown the protective effects of MyD88-targeted deletion in intestinal epithelial cells. MyD88 deletion inhibited colitis development in a model of spontaneous colitis (SHP-2 IEC-KO mice), rescued the goblet/intermediate cell ratio and prevented NFκB hyperactivation and inflammation [47]. In a model of diet-induced obesity, MyD88 KO mice exhibited increased anti-inflammatory endocannabinoids, antimicrobial peptide production, and intestinal regulatory T cells, and were partially protected against diet-induced obesity, diabetes, and inflammation [48]. Accordingly, enhanced MyD88 signalling (C57Bl/6 mice expressing a constitutively active form of TLR4 in the intestinal epithelium) resulted in an elevated bacterial translocation, impaired epithelial barrier function, different expression of antimicrobial peptide genes, and altered epithelial cell differentiation [49]. In contrast, Frantz et al. reported that a loss of epithelial MyD88 signalling caused the following effects: increased numbers of mucus-associated bacteria; the translocation of bacteria to mesenteric lymph nodes; reduced transmucosal electrical resistance; impaired mucus-associated antimicrobial activity; and, downregulated expression of polymeric immunoglobulin receptor (the epithelial IgA transporter), mucin-2 and the antimicrobial peptides RegIII and Defa-rs1. These mice were also more susceptible to experimental colitis [50]. MyD88 signalling has also been shown to stimulate gastrointestinal motility [51] and promote the differentiation of intestinal epithelial cells [52]. These rather contradictory outcomes suggest that (i) both low and excessive TLR4 signalling can promote intestinal inflammation and (ii) the interaction between the intestinal microbiota and the immune system is complex. Further research is needed in order to better understand these causal relationships. 

### 3.2. The Microbiota and Intestinal Macrophages

An important population of innate immune cells are intestinal resident macrophages, which play a central role in the maintenance of homeostasis in the gastrointestinal tract [53]. Intestinal macrophages constitutively produce IL-10, one of the major anti-inflammatory cytokines that promotes differentiation and maintenance of Treg cells. They are also hyporesponsive to TLR stimulation [54] and are less prone to the secretion of pro-inflammatory cytokines after LPS stimulation [55]. Resident intestinal macrophages contribute to the attenuation of the immune response to various bacterial and food antigens, while maintaining anti-inflammatory tone in the intestine [56,57]. Recent studies have shown that circulating and tissue-resident macrophages (lung, brain, skin, liver) consist of populations of different embryonic origin, and are also independently maintained in a steady state in adulthood [58,59]. Intestinal macrophages seem to be the only exception to this rule. During the neonatal period, intestinal macrophages are derived from embryonic precursors (the yolk sac and foetal liver), but are gradually replaced by the progeny of conventional haematopoiesis (i.e., Ly6C^hi^ blood monocytes) in adulthood [54]. This process occurs in both the small intestine and the colon, but is regulated by different mechanisms. In the colon, the establishment of the gut microbiota and commensal-derived signals [54,57] drives the replenishment of resident macrophages. Consequently, the microbiota can stimulate macrophages to produce tolerogenic IL-10 either via TLR-signalling [60,61] or via activation of intestinal macrophage receptor GPR109a by microbial metabolites (butyrate, niacin). This setting helps to establish an “immunotolerogenic” feedback loop, whereby the gut microbiota actively promotes the recruitment of circulating macrophages into the intestinal mucosa. These macrophages in turn create a tolerant environment that facilitates the growth of these bacteria. In contrast to the colon, replenishment and IL-10 production of small intestine macrophages are not regulated by the gut microbiota but directly by dietary amino acids [62]. PN administration leads to a profound decline in the number of IL-10-producing macrophages in the small intestine [62]. The compromised replenishment of IL-10-producing macrophages together with the shift in microbial composition towards a more immunogenic phenotype (TLR ligand-rich *Proteobacteria*) may act in synergy towards generating a pro-inflammatory state in the PN-dependent small intestine. To our knowledge, no data are available regarding the direct effect of PN on colon macrophages. However, the facts we do have at our disposal, namely the shift in microbiota composition and the increase in the pro-inflammatory cytokine production, at least suggest a similar scenario. 

### 3.3. The Microbiota and Paneth Cells

Paneth cells are important components of mucosal immunity. They occur at the base of small intestinal crypts and are the primary source of small peptides that exhibit antimicrobial activity (AMPs), such as secretory phospholipase A2 (sPLA2), lysozyme, and α and β-defensins [63,64,65]. Paneth cells sense bacteria via MyD88-dependent toll-like receptor (TLR) activation, which triggers antimicrobial action [66]. In the PN mouse model, the attenuated expression of Paneth cell antimicrobial proteins was associated with a compositional shift in the microbiome (decreased *Firmicutes*; increased *Bacteroidetes* and *Proteobacteria*), weaker bactericidal activity of mucosal secretions and greater susceptibility to enteroinvasion by *E. coli* [14]. Omata et al. demonstrated that PN feeding resulted in the suppressed secretion of sPLA2 and increased bacterial survival. Taking these data together, PN significantly impairs the innate immune response by suppressing Paneth cell function [67].

### 3.4. The Microbiota and B-Lymphocytes

The current paradigm supposes that the antibody response is directly or indirectly mediated through TLR signalling, and that it depends on the interaction of bacterial DNA, proteins, and cell wall components with TLR receptors [68,69]. Quite recently, Kim et al. reported a new mechanism whereby the gut microbiota affects host antibody responses via their fermentation products (SCFA) [70]. A positive correlation between dietary fibre intake and intestinal IgAs levels was shown previously [71]. Kim et al. tested the hypothesis that SCFA, the main fermentation product of dietary fibre, affects antibody production. They showed that in isolated mice spleen B-cells in vitro, SCFA increased acetyl-CoA and regulated metabolic sensors to increase oxidative phosphorylation, glycolysis and fatty acid synthesis. This in turn produced energy and building blocks that supported antibody production. In parallel, SCFA controlled gene expression and expressed molecules necessary for plasma B cell differentiation. The effects of all major SCFA (C2, C3 and C4) were comparable. In vivo, mice fed a low dietary fibre (DF) diet were more susceptible to *Citrobacter rodentium* infection compared with mice fed a high DF diet. In susceptible animals, supplementation with DF or propionate (C3) increased host resistance and the IgA response to *C. rodentium* [70]. 

## 4. PNALD and Bile Acid Metabolism

### 4.1. Bile Acid Metabolism and Function

Bile acids (BAs) are synthesised from cholesterol in the liver as primary BAs, which are represented by chenodeoxycholic (CDCA) acid and cholic (CA) acid in humans. Primary BAs are conjugated to taurine or glycine in the liver and as bile salts secreted into bile through bile-salt export proteins (BSEP, ABCB11). Approximately 95% of BAs are reabsorbed in the small intestine via enterohepatic circulation, while a minor fraction escapes before being further metabolised by the gut microbiota in the colon to the secondary BAs, such as deoxycholic (DCA), urodeoxycholic (UDCA), and lithocholic (LCA) acids in humans [72]. In rodents, muricholic (MCA) acid is synthesised as well [73]. Primary and secondary BAs fulfil multiple functions. First, they serve as physiological emulsifiers that facilitate enteral absorption of dietary fat and fat-soluble vitamins [74]. Second, they have direct antibacterial effects. Third, BAs are potent signalling molecules that regulate the host glucose and lipid metabolism as well as energy homeostasis [75,76]. This regulatory function depends on the interaction of BAs with specific receptors, predominantly farnesoid-X receptor (FXR) and G-protein-coupled receptor (TGR5) [77]. However, FXR and TGR5 have different affinities to individual BAs, as FXR is activated by CDCA ˃ DCA ˃ LCA ˃˃ CA and TGR5 is activated by LCA ˃ DCA ˃ CDCA ˃ CA [78]. 

### 4.2. Interplay Between Bile Acids and the Gut Microbiota

Because the transformation of BAs is solely dependent on the gut microbiota, the extent of BA receptor activation is largely dependent on microbiota composition [79]. FXR is a key regulator of BA metabolism. Activated FXR inhibits CYP7A1, a rate-limiting enzyme of BA synthesis in hepatocytes, by two mechanisms. In hepatocytes, FXR forms a heterodimer with SHP (small heterodimer partner) to suppress the transcription of CYP7A1 [80]. In enterocytes, activated FXR stimulates production of FGF15/19. This cytokine binds to FGFR4 receptor on the hepatocyte surface, thus suppressing CYP7A1 and BA synthesis [81]. Myiata et al. showed that antibiotic treatment elevates hepatic BA synthesis in mice via the suppression of FGF15 expression in the ileum [82], which confirms the potent effect of the gut microbiome on BA metabolism. FXR also regulates enterohepatic BA circulation by regulating the expression of BA transporters. Bacterial modifications of BAs start with primary BA deconjugation by bacteria that exhibit bile salt hydrolase (BSH) activity, followed by the formation of secondary BAs by microbes that exhibit 7α-dehydroxylase and 7α-dehydrogense activity [83]. A wide array of bacteria exhibit BSH activity, enabling them to deconjugate BAs. However, there are a few bacteria belonging to the *Clostridium* clusters XI and XVIa (exhibiting 7α-dehydroxylase activity), which may catalyse the dehydrogenation of deconjugated BAs [84]. As mentioned above, BAs are themselves toxic to bacteria. First, they have direct antibacterial effects. BAs may cause damage to the bacterial membrane because of their detergent properties [85], promote bacterial protein unfolding and aggregation [86], and trigger oxidative/nitrosative stress [87]. Second, they may influence bacterial growth indirectly via FXR activation. Among other genes, FXR controls expression of Ang1, iNos and IL18, which either counteract microbial overgrowth or protect the intestinal mucosa [88]. Activated FXR stimulates the expression of cathelicidin, an antimicrobial peptide that is active in bile ducts [89]. Nevertheless, the sensitivity of bacteria to BAs varies significantly. The growth of bacteria such as *Alistipes*, *Bilophila wadsworthia*, *Escherichia coli*, *Listeria monocytogenes* and *Bacteroides* is even facilitated by BAs, which handicap other symbiotic microbes [84]. Some bacteria are at least BA-tolerant, such as some *Lactobacillus*, *Bifidobacteria* and *Clostridium* species [87].

### 4.3. Bile Acids and PNALD

In the 1990s, Ohkohchi et al. reported that SBS children patients suffering from intractable diarrhoea exhibited a significantly increased faecal BA excretion and altered bile acid composition. They also observed that primary bile acids accounted for more than 95% of total BAs, while taurine- and glycine-conjugated BAs accounted for only 10%. In these children, the loss of bile acids was strongly associated with a decrease in the actual absorptive surface area of the residual small intestine, while growth of the normal bacterial flora was disturbed in the residual intestine [90]. The relationships between parenteral nutrition, PNALD and BA dysmetabolism were recently studied using animal models. In one SBS-PNALD model, newborn piglets were subjected to a 75% proximal small bowl resection and fed solely via the parenteral route [13]. Two and six weeks post-resection, a significant alteration in microbiota composition was observed, particularly the loss of *Clostridiales*. This was coupled with a decrease in the overall bacterial diversity in the colon as well as a shift to a primary BA-dominant profile in bile, portal serum, and colonic content. The animals also exhibited hepatic fibrosis, steatosis and inflammation. These changes were associated with a blunted FXR activation response in the intestine, altered FXR signalling in the liver and upregulated hepatic BA synthesis [83,91]. Taken together, these data suggest that the altered BA composition following microbial dysbiosis may contribute to PNALD due to the direct physiological effects of toxic BAs and altered FXR signalling.

## 5. PN and Intestinal Barrier Permeability

Epithelial barrier function is essential in order for the intestine to maintain an effective defence against intraluminal toxins, foreign antigens, and bacteria, and also for enabling the epithelium to effectively absorb nutrients. Such a defence mechanism requires an intact epithelial layer [92]. Increased incidence of sepsis is a common complication in patients dependent on long-term parenteral nutrition, and it is acknowledged that organisms arising from enteric flora constitute a large percentage of these infections [93]. In 1988, Alverdy showed that PN administration in rats significantly increased bacterial translocation to the mesenteric lymph nodes by increasing caecal bacterial counts and impairing the intestinal defence [94]. In subsequent years, the loss of epithelial barrier function was identified as one of the key factors in the development of septic complications associated with long-term PN dependence [95]. PN-associated loss of the epithelial barrier function is probably the result of several underlying mechanisms. First, the morphology of the intestinal wall is altered and the functionality of epithelial cells is compromised. Studies performed using PN mouse models have revealed structural changes, including the atrophy of small bowel villi, an increase in epithelial cell apoptosis and a decrease in epithelial cell proliferation [96,97,98]. Second, PN administration is associated with increased production of pro-inflammatory cytokines [99,100], while in vitro studies have demonstrated that cytokines produced by immune cells can result in increased permeability of the intestinal mucosa [101]. In particular, the upregulation of IFNγ and TNFα expression in intraepithelial lymphocytes together with decreased production of IL-10 have been identified as the main factors contributing to this pathology [92,102]. Finally, tight junction proteins have an essential role in the maintenance of epithelial barrier function. Among them, ZO (1 and 2), claudins and occludins are the most important and critical components in the structural and functional organisation of tight junctions [103,104,105]. These proteins regulate the transport of ions and small proteins across the intestinal wall, while their expression is downregulated with PN [92]. Increased TNFα signalling due to PN-associated pro-inflammatory status results in the dissociation of structural protein ZO-1 from tight junctions and worsens barrier function [106]. The exact mechanism underlying the aggravation of epithelial barrier function has not been fully elucidated yet, but there is strong evidence to suggest that an alteration in TLR signalling may represent a link between PN-induced changes in intestinal permeability and changes in microbiota composition. 

It is important to stress that all of the above results have been obtained using PN models combined with complete enteral nutrient deprivation. The deprivation of enteral nutrients available to the intraluminal bacteria alters the selection pressure for determining the dominant species in the microbiota. In environments of relative starvation, *Proteobacteria* tend to dominate [107], while *Firmicutes* are usually the predominant group in enterally fed states [108]. Wildhaber et al. [97] showed that limited enteral feeding (covering only 25% of total caloric requirements), completely reversed the unfavourable phenotype associated with parenteral nutrition (increased bacterial translocation, elevated production of pro-inflammatory cytokines, T-subpopulation representation in the intestinal epithelium). Unfortunately, the authors did not determine microbiota composition, so it is not possible to discern whether the beneficial effect arose only from the provision of nutrients to intestinal epithelial cells or whether it was co-associated with a shift in microbiota composition.

## 6. PN and Pro/Prebiotic Treatment

We recently showed that the microbiota of adult SBS patients dependent on total parenteral nutrition is depleted of anaerobic butyrate producers and that the amount of SCFA (particularly propionate and butyrate) in luminal content is accordingly very low [32]. Intestinal dysbiosis is often treated with probiotics, which are defined as live microorganisms that confer a health benefit on the host when administered in adequate amounts [108]. Positive results have been reported for probiotic use in the management of postsurgical inflammatory bowel disease [109,110], antibiotic-associated diarrhoea [111], and necrotising enterocolitis [112]. The most popular components of probiotic preparations are the members of the *Lactobacillus* and *Bifidobacterium* genera, and their usage is usually safe. Nevertheless, in cases where probiotics have been administered to SBS children patients, positive as well as adverse effects have been reported. The only available double-blind, placebo-controlled randomised crossover clinical trial focusing on the effects of probiotics (*Lactobacillus rhamnosus* LCG) on intestinal permeability in SBS children was unable to prove any beneficial or negative effects [113]. A case-control study performed by Uchida et al. [114] evaluated the effects of treatment on *Bifidobacterium breve*, *Lactobacillus casei* and galacto-oligosaccharides. They proved elevation of stool SCFA in 3 out of 4 patients as well as a trend for increases in height and weight velocity. Five of the nine case studies delivered evidence on the positive effects of probiotic supplementation, while the remaining four reported adverse effects, such as *Lactobacillus* sepsis and d-lactic acidosis [115]. The variable outcomes of probiotic intervention are not surprising, bearing in mind the prevalence of *Lactobacilli* in the SBS microbiome. SBS patients suffer, not because of the lack of lactate-producers, but because of the virtual absence of butyrate-producers that use lactate in the healthy gut. In this setting, further supplementation with lactate-producing bacteria or their substrates (indigestible oligosaccharides) would not provide any benefit, and, with respect to microbiota composition in some cases, could even be detrimental. Supplementation of strictly anaerobic butyrate producers such as *Lachnospiraceae*, *Ruminococcaceae* and others could be theoretically more effective, but because of the significantly altered short-bowel environment (higher oxygen levels, more acidic pH, shorter transit time) the effectiveness of this treatment would be questionable. An interesting option for bypassing all of these obstacles is to supplement SCFA directly to the nutrition mixture. Using neonatal piglets that had undergone 80% proximal jejunoileal resection, Bartholome et al. demonstrated that the supplementation of a mixture with SCFA (acetate, propionate, and butyrate) or butyrate alone enhanced structural adaptations in the developing intestine [116]. In a mouse model, PN enriched with butyric acid partially reversed the parenteral nutrition-associated atrophy of gut-associated lymphoid tissue and improved IgA secretion in the intestinal and extraintestinal mucosae. In addition, it moderately recovered mucosal atrophy [117]. Other studies report that the intravenous butyrate improves the mechanical strength of colonic anastomosis [118], moderately increases mucosal protein synthesis [119] and ameliorates small-intestinal mucosal atrophy (PN rat model) [120]. Taken together, these data advocate intravenous SCFA (particularly butyrate) supplementation as a new option for the treatment of PN-associated intestinal complications.

## 7. Role of the Microbiota in PNALD Development

**Figure 1:**
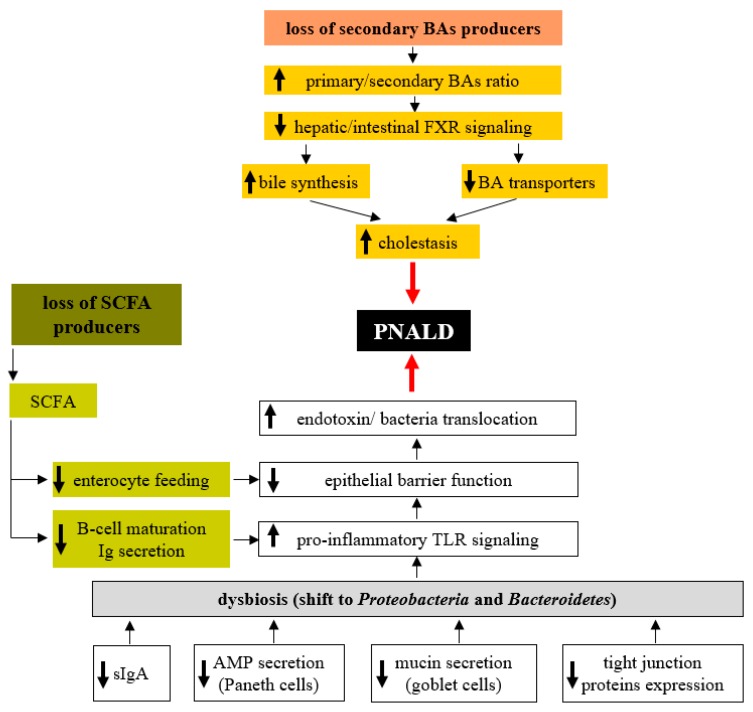
Gut microbiota-related factors contributing to PNALD development. Administration of parenteral nutrition (PN) is associated with decreased production of sIgA, reduced mucin synthesis in goblet cells and impaired antimicrobial function of Paneth cells. All these factors favour the growth of pathogenic bacteria (mostly from *Proteobacteria*) at the expense of beneficial commensals. In addition, the reduction in sIgA enables greater microbial access to the host epithelium and triggers an inflammatory response in the lamina propria. Enhanced toll-like receptors (TLR)-signalling due to the increased presence of potential pathogens stimulates the synthesis of pro-inflammatory cytokines in immune cells. Altogether, these factors contribute toward compromised epithelial barrier function (EBF) and increased translocation of endotoxins and even whole bacteria to the portal circulation and the liver, thereby inducing the inflammatory response. The lower abundance of SCFA producers results in decreased short-chain fatty acids (SCFA) availability, which attenuates B-cell maturation, specific antibody production and increased susceptibility to pathogens. The specific loss of secondary bile acids (BA) producers (*Clostridiales*) results in a significant shift towards primary BAs in faeces and impaired hepatic and intestinal farnesoid-X receptor (FXR) signalling. Consequently, bile acid synthesis in the liver becomes upregulated and expression of bile acid transporters becomes downregulated, resulting in the attenuation of BA transport to bile and the development of cholestasis.

In the previous sections, we discussed the essential role of the gut microbiota in the maintenance of homeostasis in the intestinal environment and the adverse effects of dysbiosis (Figure 1). Recent results suggest that the overall decrease in microbial diversity and the overgrowth of the specific bacterial groups in the colonic microbiota are associated with PNALD. Wang et al. [121] showed that gut microbiota composition in SBS infant patients reflects the occurrence of complications like PNALD or central line-associated bloodstream infection (CLABSI). Although the overall diversity and number of bacterial species in samples from the asymptomatic group was similar to those from healthy control infants, there were striking differences in children suffering from PNALD and CLABSI. In addition to the decrease in diversity and the number of bacterial species, there was a shift from Gram-positive *Firmicutes* to Gram-negative *Proteobacteria* (mainly *Enterobacteriaceae*). Many *Proteobacteria* produce bacterial lipopolysaccharide (LPS), a potent hepatotoxic compound, and prolonged exposure to higher LPS concentrations may result in liver injury. Furthermore, the microbiome, when enriched in *Proteobacteria,* was demonstrated to accelerate liver fibrogenesis, which may contribute to PNALD development [122]. El Kasmi et al. demonstrated the interplay between intestinal injury and the intestinal microbiota in the development of PNALD. In mice, intestinal injury and increased permeability were induced by short-term (4 days) oral treatment with low doses of dextran sulphate sodium (DSS), followed by the continuous infusion of a soy lipid-based PN solution for 7 or 28 days. After 7 days on PN, the mice showed an increased intestinal permeability, elevated portal vein LPS levels, evidence of hepatocyte injury (elevated serum aspartate aminotransferase, alanine aminotransferase), cholestasis (elevated serum bile acids, total bilirubin), and an increased expression of interleukin-6, tumour necrosis factor alpha and transforming growth factor beta in Kupffer cells. Markers of liver injury remained elevated and were associated with lobular inflammation, hepatocyte apoptosis, peliosis and Kupffer cell hypertrophy and hyperplasia after 28 days on PN [123]. Interestingly, PN infusion without DSS pretreatment or DSS pretreatment alone did not result in liver injury or Kupffer cell activation. Suppression of the intestinal microbiota with broad-spectrum antibiotics and ablation of TLR4 signalling in *Tlr4* mutant mice prevented PNALD development, which strongly suggests the role of the microbiota in its etiopathogenesis. In a subsequent study, Harris et al. identified the specific microbial taxa associated with PNALD development in this model. Among those, members of the *Erysipelotrichaceae* family and representatives of the Gram-negative S24-7 lineage of *Bacteroidetes* were identified as candidates that might play a causal role in the pathogenesis of PNALD [124]. Interestingly, mice treated only with PN without intestinal injury did not develop PNALD, despite the similar composition of their intestinal microbiota. Taken together, these experiments suggest that at least two independent conditions must be met concurrently; (i) overgrowth of specific bacteria due to PN administration and (ii) increased intestinal permeability allowing MAMPs (microbe-associated molecular pattern) derived from these taxa to reach the liver and subsequently activate Kupffer cells [124,125]. 

## 8. Conclusions

A growing amount of evidence suggests that the deterioration of hepatic function in conjunction with long-term PN dependence is not the consequence of PN administration *per se* but because of intestinal failure and associated complications. The prominent factors seem to be increased permeability of the intestinal barrier, which facilitates massive translocation of bacterial toxins and even microorganisms into the portal circulation, mesenteric lymph nodes and liver, and overall pro-inflammatory status in the compromised intestine. The gut microbiota plays a profound role in the maintenance of intestinal barrier function and the establishment of either an immunotolerant or inflammatory setting of intestinal immunity. Therapeutic strategies focus on microbiota composition itself through the targeted delivery of beneficial microbiota products or by supplementation with immunomodulators. Nevertheless, understanding the complex interactions between the gut microbiota and the modified intestinal environment in PN patients is a crucial condition for efficient treatment. 

## Figures and Tables

**Table 1:** The effect of parenteral nutrition on gut microbiome composition.

Model	Bowel Resection	TPN Duration	Enteral Feeding		References
Rat-adult	No	14 days	No	*Firmicutes/Bacteroidetes* ratio; shift in favour of Bacteroidetes	[10]
Mouse-adult	No	5 days	No	Shift from *Firmicutes* to *Proteobactoria* and *Bacteroidetes*, i.e., *Salmonella*, *Proteus*, *Escherichia*, *Bacteroides*	[11]
Mouse-adult	No	5 days	No	Shift from *Firmicutes* to *Bacteroidetes* and *Proteobacteria*	[12]
Piglet-newborn	Yes	6 weeks	No	Changes in the composition of the *Firmicutes* phylum (decrease of *Anaerotruncus*, *Clostridium*, *Ruminococcus*, *Peptostreptococcus*; increase in *Acidaminococcus* and *Mitsuokella*)	[13]
Piglet-newborn	No	7 days	No	Lower total bacterial counts and reduced bacterial diversity, enriched in *Clostridium difficile*	[14]
Piglet-newborn	No	7 days	No	Enriched in *C. perfringens* and sulphated monosaccharide-degrading bacteria	[15]
Piglet-newborn	No	14 days	No	PN + ω-3: increased *Parabacteroides*	[16]
				PN + ω-6: increased *Enterobacteriaceae*	
Human-pre-term newborn	Yes	Long-term	Yes	Higher diversity, higher abundance of Gram-negative bacteria, lower odds of death and late-onset sepsis cases	[17]
			No	Less diversity, lower abundance of Gram-negative bacteria, increased odds of death and late-onset sepsis cases	
Human-paediatric/adult	Yes	Long-term	Yes	Increased *Staphylococcus*, *Pseudomonas*, *Campylobacter*, *Propionibacterium*, *Chryseomonas*	[18]
			No	Increased *Enterobacter*, *Shigella*, *Klebsiella*, *Fusobacterium*	
Human-adult	Yes	Long-term	Yes	Enrichment in *Lactobacillus/Leuconostoc*; depletion of anaerobes, especially *Clostridiaceae*	[19,20]
Human-adult	Yes	Long-term	Yes	High abundance of *Proteobacteria*, especially *Enterobacteriaceae* and *Fusobacteria*; changes in the *Firmicutes* spectrum, depletion of *Lachnospiraceae* and *Ruminococcaceae*	[21]
